# Clinical, microbial and metabolic characteristics of gas-forming pyogenic liver abscess and its potential formation mechanism

**DOI:** 10.3389/fcimb.2026.1649624

**Published:** 2026-02-11

**Authors:** Zibo Gong, Yawen Guo, Hongguang Wang, Lulu Chen, Hairui Wang, Zhihui Chang

**Affiliations:** Department of Radiology, Shengjing Hospital of China Medical University, Shenyang, China

**Keywords:** 16S rDNA sequencing, gas formation, pyogenic liver abscess, risk factor, untargeted metabolomics

## Abstract

**Introduction:**

Pyogenic liver abscess (PLA) is a common intra-abdominal infection with substantial morbidity and mortality, among which gas-forming pyogenic liver abscess (GFPLA) represents a more severe clinical subtype. Basically, GFPLA is usually associated with more severe clinical outcomes compared to non-gas-forming PLA (non-GFPLA). The underlying mechanisms driving gas formation remain unclear. This study aimed to explore clinical, microbial, and metabolic characteristics of GFPLA.

**Materials and methods:**

A total of 176 PLA patients (39 GFPLA, 137 non-GFPLA) were retrospectively analyzed. Clinical variables were compared between groups. Pus samples collected from patients undergoing percutaneous drainage were analyzed using 16S rDNA sequencing and untargeted metabolomics to investigate microbial composition and metabolic differences.

**Results:**

GFPLA patients showed worse liver function, higher inflammatory markers, poorer glycemic control, and higher in-hospital mortality. 16S rDNA sequencing revealed no significant differences in bacterial richness, diversity, or community composition between groups, with *Klebsiella* dominating in both. Functional microbial predictions showed no association with gas formation. Untargeted metabolomics identified distinct metabolic profiles in GFPLA, with key differential metabolites positively correlated with blood glucose and inflammatory markers but not with *Klebsiella* abundance.

**Conclusion:**

Diabetes mellitus is an independent risk factor for GFPLA. Gas formation in PLA is more likely linked to high blood glucose-induced metabolic alterations in the liver micro-environment rather than microbial composition. These findings suggest new potential therapeutic targets by modulating metabolic pathways to improve GFPLA outcomes.

## Introduction

Pyogenic liver abscess (PLA) is a common and potentially life-threatening intra-abdominal infectious disease with an increasing incidence worldwide, accounting for approximately 80% of all liver abscesses and associated with an in-hospital mortality rate of about 5.7% (Jepsen et al., 2005; Webb et al., 2014; Yin et al., 2021; Cui et al., 2023). Gas formation is a common imaging feature of PLA with a reported incidence of 7%–24% (Yin et al., 2021; Chan et al., 2022). Studies have demonstrated that patients with gas-forming pyogenic liver abscess (GFPLA) tend to have more severe symptoms, longer hospital stay, and higher mortality rates than patients with non-GFPLA (Tee Yu et al., 2017; Thng et al., 2018; Zhang et al., 2021). Although PLA is frequently associated with diabetes mellitus, hepatobiliary disorders, and immunocompromised states, the mechanisms underlying gas formation in GFPLA remain poorly understood. Elucidating these mechanisms may provide important insights into disease progression and inform the development of targeted therapeutic strategies to improve patient outcomes.

In clinical practice, gas formation is sometimes considered a distinct pathological stage in the progression of PLA. It has been demonstrated that gas within the abscess cavity in PLA mainly originates from two pathways: retrograde translocation of intestinal gas secondary to biliary tract disruption (Farrugia and Szurszewski, 2014; Linden, 2014), which has been talked much, and intralesional gas production by microorganisms inside the pus cavity (Khan et al., 2015; Kwon et al., 2018). K*. pneumoniae* is widely regarded as the most common causative pathogen of pyogenic liver abscess (Chan et al., 2022; Cui et al., 2023). While gas-forming pyogenic liver abscess has been reported in association with gas-forming bacteria such as *Clostridium perfringens* (*C. perfringens*) and *Salmonella enteritidis*(*S. enteritidis*) (Tee Yu et al., 2017; Stebner et al., 2021), increasing evidence suggests that *K. pneumoniae* remains the most prevalent pathogen in GFPLA (Altaie et al., 2021; Bivand et al., 2021). However, no studies have thoroughly investigated the relationship between the microbial composition within the abscess cavity and the development of GFPLA.

In recent years, the rapid advancement of high-throughput sequencing technologies has established 16S rDNA gene sequencing and untargeted metabolomics as powerful approaches for characterizing complex microbial communities and metabolic profiles (Smirnova et al., 2020; Altaie et al., 2021; Bivand et al., 2021; Moon et al., 2021; Stebner et al., 2021). When combined, these methods not only enable comprehensive analysis of the microbial composition and metabolic state within the PLA abscess cavity, but also reveal potential interactions between them, thereby contributing to a deeper understanding of the underlying mechanisms involved in GFPLA formation.

In this study, untargeted metabolomics and 16S rDNA sequencing were employed to analyze the metabolic profiles and pathogenic bacterial composition within the abscess cavity, with the aim of elucidating the characteristics of gas-forming pyogenic liver abscess (GFPLA) and the potential mechanisms underlying its formation.

## Materials and methods

### Study population

This retrospective study continuously collected data from patients diagnosed with PLA between January 2018 and December 2022. The medical records of eligible patients were thoroughly reviewed. Eligible patients were assigned to the GFPLA or non-GFPLA group based on the presence or absence of gas on preoperative CT. Additionally, pus samples were prospectively collected for 16S rDNA sequencing and untargeted metabolomics analysis from patients with PLA who underwent percutaneous abscess drainage between January 2022 and December 2022. The Ethics Committee of Shengjing Hospital of China Medical University approved this retrospective study (2022PS1067K).

The diagnostic criteria for PLA are as follows: (1) the patient exhibits symptoms such as fever, chills, nausea, and abdominal discomfort; (2) abdominal imaging indicates the presence of PLA; (3) bacterial culture results are positive, or there is an effective response to antibiotic treatment; (4) PLA is confirmed by percutaneous liver abscess aspiration or surgical intervention; (5) other conditions such as amebic or tuberculous liver abscess are ruled out. A diagnosis of PLA is confirmed if criteria 1 and 2 are met, along with at least one of criteria 3, 4, or 5.

The exclusion criteria for collecting clinical data are: (1) no available preoperative CT images; (2) no conventional culture results; (3) patients had a history of biliary abnormality. Biliary abnormality included a history of cholecystenterostomy, sphincteroplasty of the ampulla of Vater, and biliary stent placement.

### Clinical variables

The following information was recorded from the patients’ clinical records: demographic data; presence of DM; time from symptom onset to CT; laboratory data; hospital stay; in-hospital mortality; and conventional culture results. The time from symptom onset to CT was measured from the first appearance of PLA symptoms (e.g., fever, chills, right upper abdominal pain, abdominal discomfort, nausea, vomiting, loss of appetite) to the first CT after hospital admission.

### 16S rDNA sequencing

The full details of the microbiome methodology are provided in the **Supplementary A1**. Length heterogeneity PCR fingerprinting was routinely applied to rapidly survey our samples and standardize the community amplification. The microbial taxa associated with the pus microbiome was characterized by multi-tag sequencing. Statistical analysis was performed using the obtained feature table and feature sequence, as described in the Supporting Information. Alpha and beta diversity were calculated using QIIME2, and all graphs were constructed using the R package (R Foundation for Statistical Computing, Vienna, Austria). The alpha diversity indices included the Chao1 index (indicating bacterial community richness), the Shannon and Simpson indices (indicating bacterial community diversity), and the Pielou-e index (indicating bacterial community evenness). The beta diversity included principal component analysis, principal coordinates analysis and so on.

### Untargeted metabolomics analysis

The full details regarding untargeted metabolomics analysis are provided in **Supplementary A2**. Here, we briefly describe the sample analysis. Partial Least-Squares Discriminant Analysis (PLS-DA) was conducted using metaX to discriminate the different variables between groups and the variable importance for the projection (VIP) value was calculated. A VIP cut-off value of 1.0 was used to select important features. A volcano plot was used to compare differential metabolites between the two groups. Kyoto Encyclopedia of Genes and Genomes (KEGG) enrichment analysis was performed on significantly different metabolites (satisfying ratio≥2 or ratio ≤ 0.5; a value of p ≤ 0.05; VIP>1). All graphs were constructed using the R package (R Foundation for Statistical Computing).

### Statistical analysis

Continuous variables are presented as mean ± standard deviation or median (interquartile range), as appropriate. Categorical variables are presented as n (%). Differences in continuous variables were assessed using Student’s *t* test or the Mann–Whitney *U* test, depending on data distribution. Categorical variables were compared using the chi-squared test or Fisher’s exact test, as appropriate. Correlation analyses were conducted using Pearson or Spearman correlation coefficients based on data characteristics. All statistical analyses were performed using SPSS software, version 26.0 (IBM Corp., Armonk, NY, USA). A two-tailed *P* value < 0.05 was considered statistically significant. Graphs and figures were generated using GraphPad Prism, version 8.0 (GraphPad Software, San Diego, CA, USA).

## Results

### Characteristics of the study population

**Figure 1:**
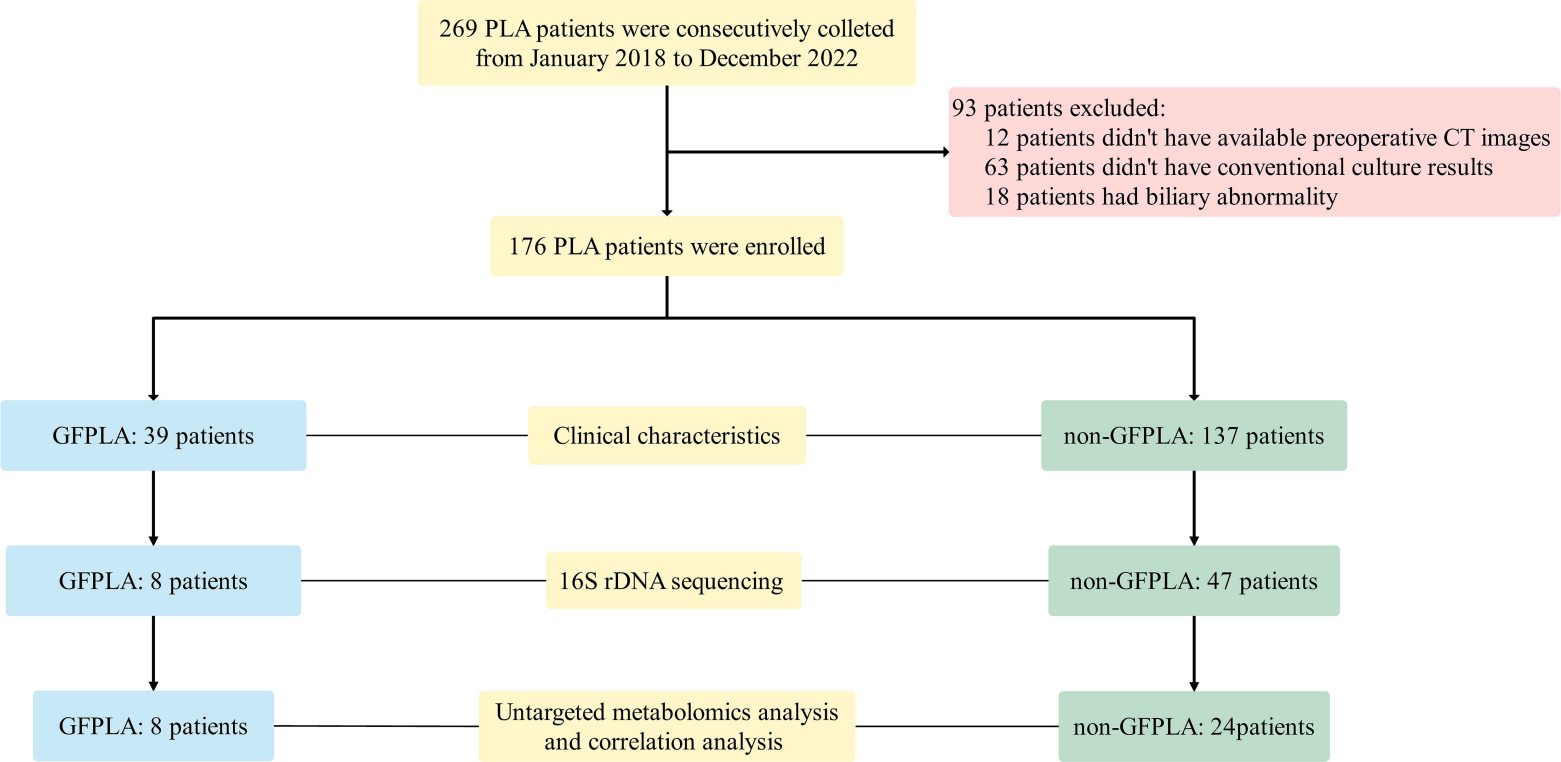
Workflow of the present study.

The workflow of this study is illustrated in **Figure 1**. A total of 269 patients were initially identified during the study period, of whom 176 were enrolled in the final analysis (GFPLA, n = 39, 22.2%; non-GFPLA, n = 137, 77.8%). The proportion of patients with DM was significantly higher in the GFPLA group (P < 0.001). Additionally, platelet counts (P = 0.034) and albumin levels (P < 0.001) were significantly lower in the GFPLA group, while levels of aspartate aminotransferase (P = 0.033), alkaline phosphatase (P = 0.036), fasting blood glucose (P < 0.001), glycated hemoglobin A1c (P = 0.003), and C-reactive protein (P = 0.040) were significantly higher. These findings suggest that patients with GFPLA exhibited poorer liver function, suboptimal glycemic control, and more pronounced inflammatory responses. The in-hospital mortality rate was also significantly higher in the GFPLA group (P = 0.009), whereas the time from symptom onset to computed tomography (CT) (P = 0.621) and length of hospital stay (P = 0.645) did not differ significantly between the two groups.

Besides, the conventional culture results showed that *K. pneumoniae* was the most common pathogen in both GFPLA and non-GFPLA groups, but all the results of conventional culture showed no significant differences between two groups (P all >0.05). Detailed characteristics of the patients are summarized in **Table 1**.

**Table 1:** Clinical characteristics of 176 patients with PLA.

Characteristics	GFPLA (n = 39)	non-GFPLA (n = 137)	P value
Male, n (%)	21 (53.80)	75 (54.70)	0.921
Age, years	59 (52–66)	60 (47–69)	0.895
DM, n (%)	34 (87.20)	64 (46.70)	<0.001
Time from symptom onset to CT, days	7 (4–15)	8 (5–14)	0.621
Leukocytes, × 10^9^/L	10.80 (8.00–15.00)	10.71 (8.12–13.75)	0.747
PLT, × 10^9^/L	220 (100–320)	257 (145–364)	0.034
ALB, g/L	26.85 (24.15–29.55)	29.90 (26.95–33.50)	<0.001
TBIL, µmol/L	11.30 (8.75–19.48)	11.80 (8.80–19.55)	0.942
ALT, U/L	46 (25–116)	31 (20–58)	0.033
AST, U/L	40 (23–76)	31 (20–58)	0.269
ALP, U/L	194 (124–277)	150 (106–230)	0.036
PT, s	13.80 (12.70–15.40)	13.80 (12.78–14.90)	0.868
FIB, g/L	4.90 (3.88–5.55)	5.20 (4.58–5.71)	0.146
DD, µg/L	1070(632–3533)	1090 (581–2109)	0.289
FBG, mmol/L	11.94 (8.14–15.30)	6.94 (5.73–11.09)	<0.001
HbA1c(%)	10.70(8.70-12.40)	7.50(6.05-10.80)	0.003
CRP, mg/L	151.00(106.50-293.43)	128.65(72.23–193.75)	0.040
In-hospital mortality, n (%)	4 (10.30)	1 (0.70)	0.009
Hospital stay, days	10.5 (7–19.5)	10 (7–14)	0.645
Conventional culture results			
*K. pneumoniae*, n(%)	31(79.49)	116(84.67)	0.137
*E. coli* ,n(%)	3(7.69)	3(2.19)	0.096
*E. faecalis* ,n(%)	1(2.56)	0(0.00)	0.222
Others, n(%)	2(5.13)	5(3.65)	0.652
Negative, n(%)	2(5.13)	13(9.49)	0.527

Data are presented as median (interquartile range) unless otherwise indicated.

DM, diabetes mellitus; CT, computed tomography; NEUT, neutrophils; LY, lymphocytes; PLT, platelets; ALB, albumin; TBIL, total bilirubin; ALT, alanine aminotransferase; AST, aspartate transaminase; ALP, alkaline phosphatase; GGT, gamma-glutamyl transferase; PT, prothrombin time; FIB, fibrinogen; DD, D-dimer; FBG, fasting blood glucose; HbA1c, Glycated hemoglobin A1c; CRP, C-reactive protein *K. pneumoniae*, *Klebsiella pneumoniae*; *E. coli* , *Escherichia coli*; *E. faecalis* , *Enterococcus faecalis*.

### 16S rDNA sequencing revealed that alpha diversity and bacterial community composition were similar between the groups

**Figure 2:**
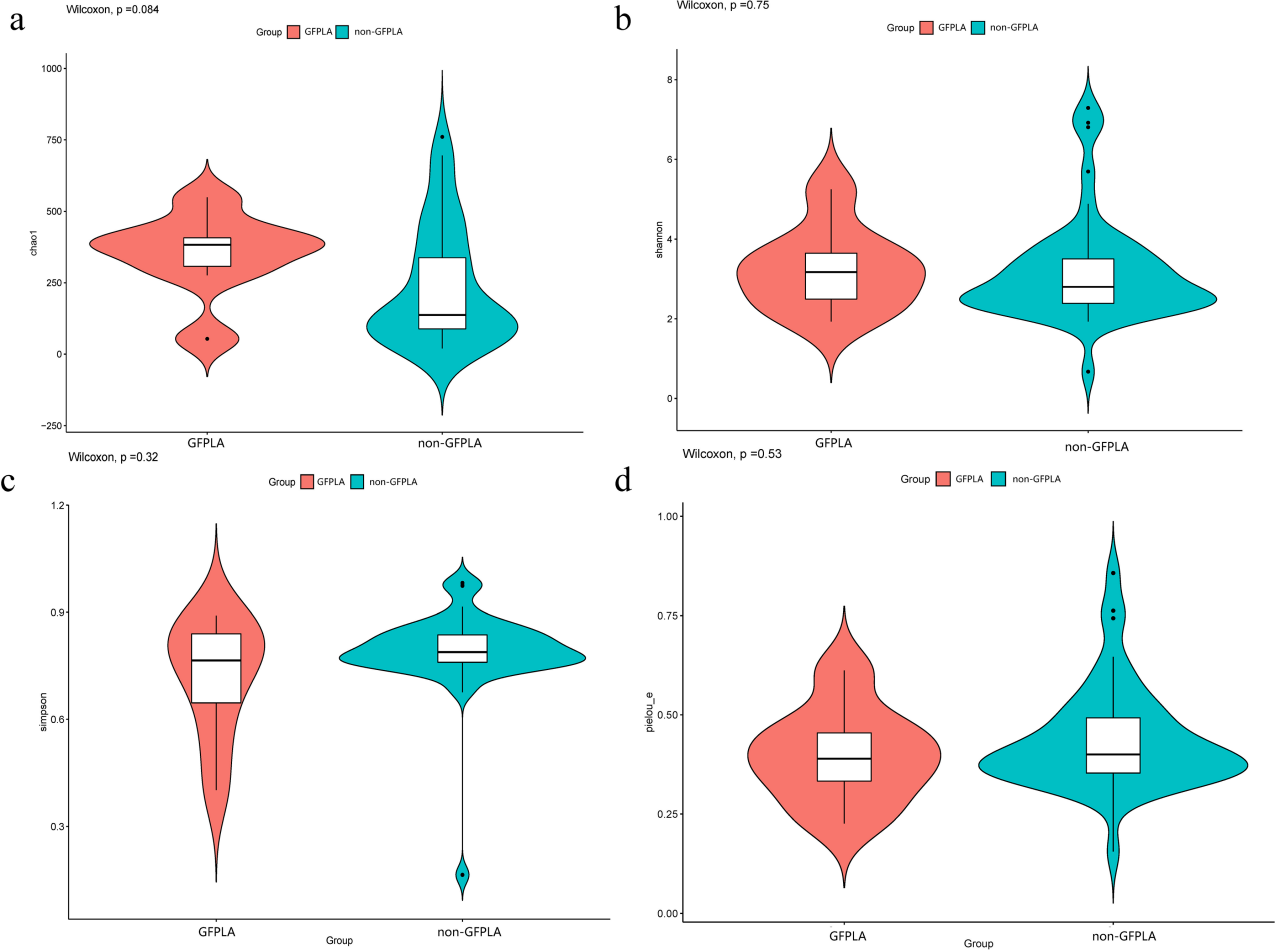
Alpha diversity did not differ between the gas-forming pyogenic liver abscess (GFPLA) and non-GFPLA groups. **(a)** The Chao1 index revealed similar bacterial community richness between the groups. **(b, c)** The Shannon and Simpson indices revealed similar bacterial community diversity between the groups. **(d)** The Pielou-e index revealed similar bacterial community evenness between the groups.

**Figure 3:**
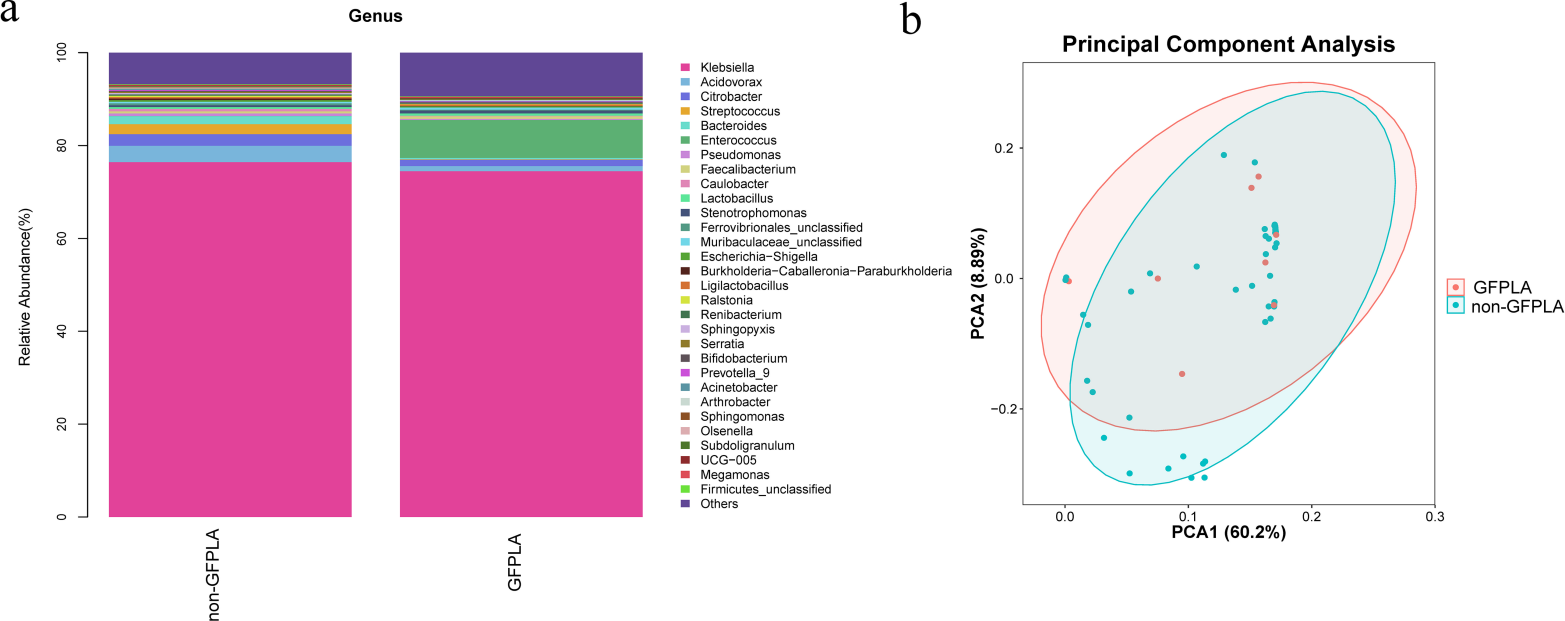
Bacterial community composition analysis and principal component analysis (PCA) of the gas-forming pyogenic liver abscess (GFPLA) and non-GFPLA groups. **(a)** Bacterial community composition analysis at the genus level illustrated the top 30 pathogenic bacteria by relative abundance. The results revealed that Klebsiella was the most common pathogen in both groups. **(b)** The PCA plot revealed that the bacterial composition of the two groups was similar.

16S rDNA sequencing was performed using 55 pus samples from patients with PLA (GFPLA, n = 8; non-GFPLA, n = 47) to explore differences in bacterial community between the GFPLA and non-GFPLA groups. Alpha diversity indices (Chao1, Shannon, Simpson, and Pielou-e) did not differ between the groups, suggesting that bacterial community richness, diversity, and evenness were similar between the groups (**Figures 2a–d**). Bacterial community composition analysis at the genus level (**Figure 3a**) illustrated the top 30 pathogenic bacteria by relative abundance, and the results revealed that *Klebsiella* was the most common pathogen in both groups. The GFPLA and non-GFPLA groups could not be adequately separated according to the principal component analysis (PCA) plot (**Figure 3b**), indicating that the bacterial composition of the two groups was similar. The further correlation analysis among pathogenic bacterial genera (**Supplementary Figure S1**) shows that *Klebsiella* generally has a negative correlation trend with other genera, suggesting that as the main dominant genus, *Klebsiella* may inhibit the growth trends of other genera.

### Prediction of microbiome phenotypes and bacterial functional potential in GFPLA and non-GFPLA groups

**Figure 4:**
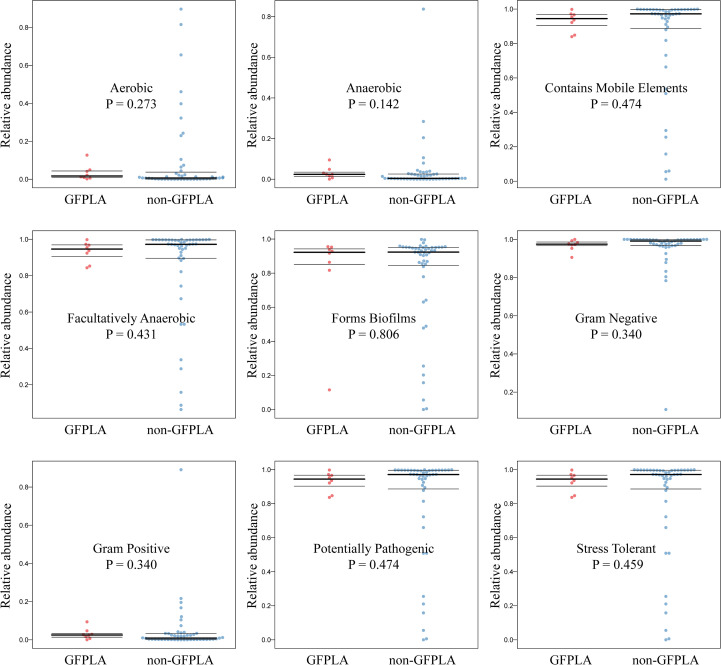
Prediction of microbiome phenotypes (aerobic, anaerobic, contains mobile elements, facultatively anaerobic, forms biofilms, gram negative, gram positive, potentially pathogenic and stress tolerant) in GFPLA and non-GFPLA groups, and there were no significant differences in all microbiome phenotypes between GFPLA and non-GFPLA groups.

As shown by the potential prediction of phenotypic function in the bacterial community in pus samples (**Figure 4**), nine potential microbial phenotypes were detected. However, there were no significant differences in biofilm forming, gram negative, potentially pathogenic, gram positive, stress tolerant, anaerobic, aerobic, facultatively aerobic and mobile element containing (P all> 0.05), which indicated that these functions may not be affected by the formation of gas in the cavity. The data from the Cluster of Orthologous Groups (COG) database were further analyzed using the PICRUSt program based on high-throughput sequencing data of 16S rDNA and the enrichment of metabolic pathways is observed in **Supplementary Figure S2**. Among these differential metabolic functional characteristics, the relative abundance of “Glutathione peroxidase, house-.lipid peroxides” pathway was significantly higher than other metabolic functional categories.

### Untargeted metabolomics revealed that there were significant differences in metabolites and metabolic pathways between the two groups, and some of the differential metabolites are closely related to blood glucose levels

**Figure 5:**
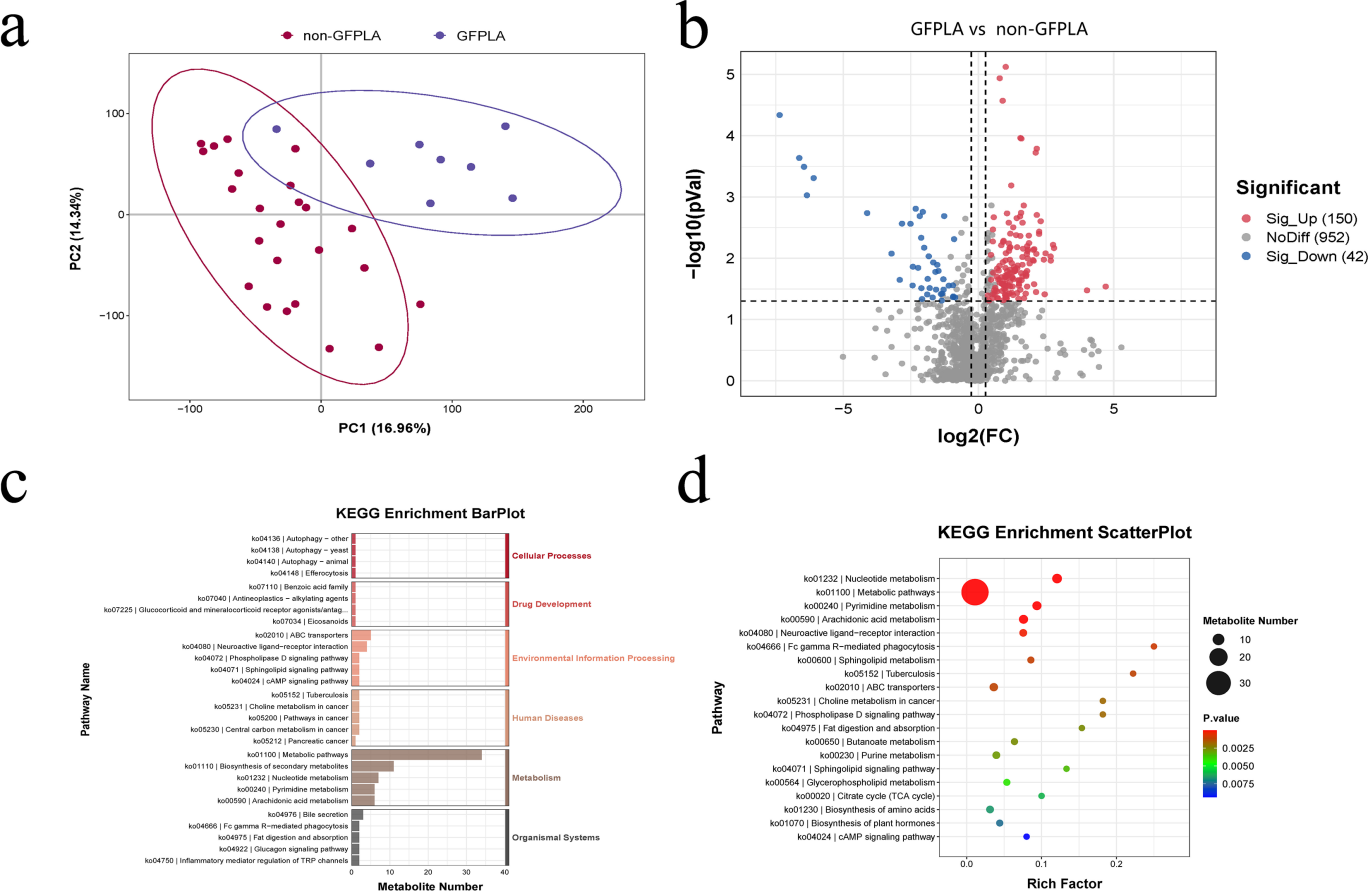
Untargeted metabolomics results of the GFPLA and non-GFPLA. **(a)** PLS-DA revealed significant metabolic differences between the two groups. **(b)** Volcano plot showed there were 192 differential metabolites between the two groups. **(c, d)** Selected examples of KEGG pathway enrichment.

An untargeted metabolomic analysis was conducted on 32 pus samples(GFPLA: n = 8; non-GFPLA: n = 24) to investigate the metabolic characteristics within the abscess cavity. PLS-DA revealed significant metabolic differences between the two groups (**Figure 5a**). To sum up, we identified, based on the KEGG database, 192 metabolites that differed in abundance between the two groups, including both host-derived and bacterially derived metabolites (**Figure 5b**). To further explore the functional mechanism of the differential metabolites, the pathway enrichment analysis using KEGG database was carried out (**Figures 5c, d**). The results showed that differently expressed metabolomics in patients with GFPLA were most significantly enriched in metabolic pathways, followed by biosynthesis of secondary metabolites, nucleotide metabolism, pyrimidine metabolism, arachidonic acid metabolism and ABC transporters(In each of the aforementioned pathways, the number of enriched metabolites is greater than or equal to 5).

**Figure 6:**
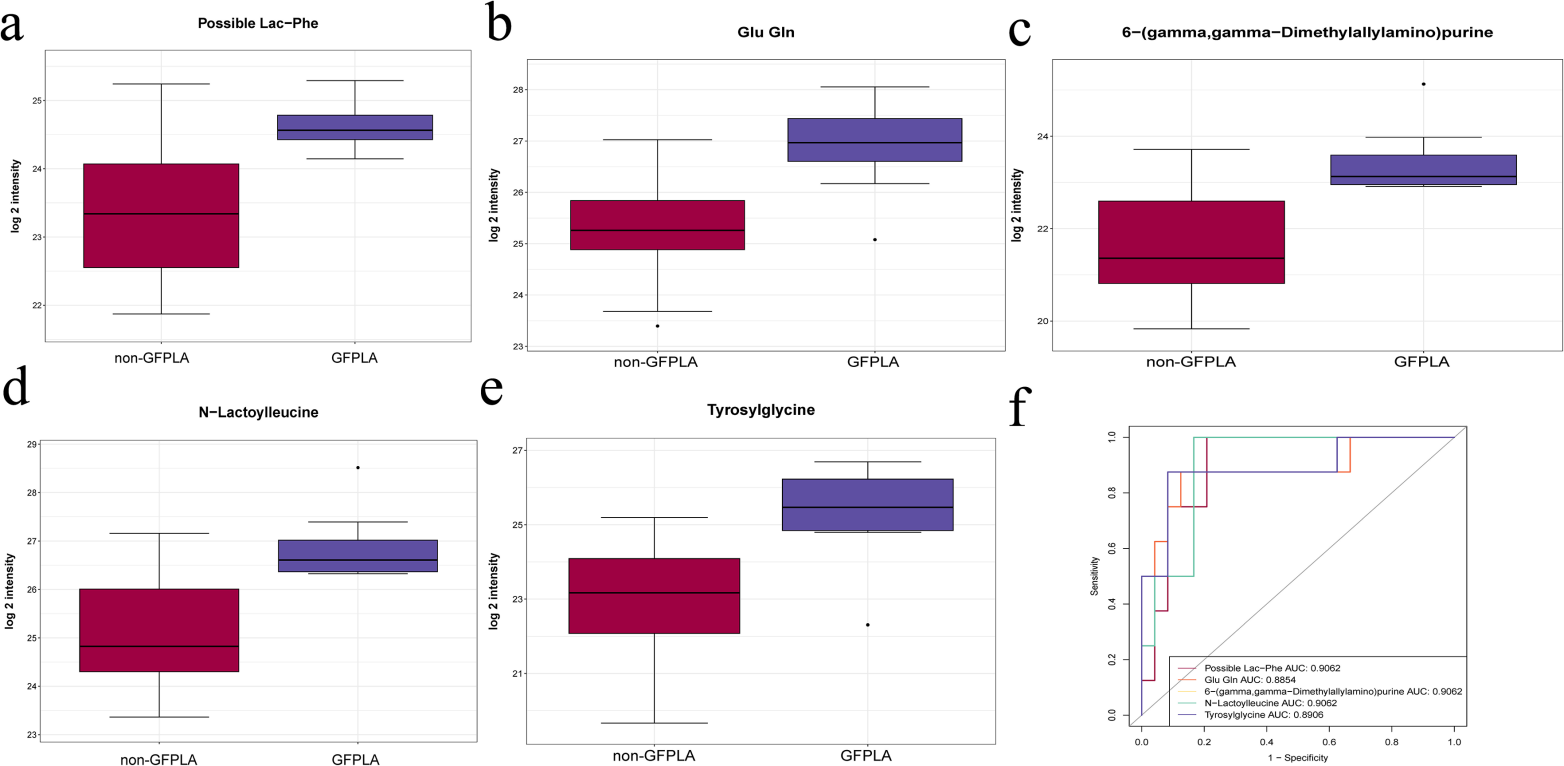
Untargeted metabolomics results of the GFPLA and non-GFPLA. **(a–e)** The five most significantly different metabolites between the two groups. **(f)** Receiver operating characteristic curve analysis of the five most significantly different metabolites between the two groups.

The metabolites that were altered significantly were selected according to a variable VIP score of the PLS model(VIP≥1) and the P values from t tests(P value<0.05). The current study displayed the five most significantly different metabolites (possible lac−phe, glu gln, 6−(gamma,gamma−dimethylallylamino)purine, N−lactoylleucine and tyrosylglycine), which are all enriched in the GFPLA group (**Figures 6a–e**). Receiver operating characteristic curve (ROC)analysis was further performed to quantify the diagnostic performance of quantitative metabolites. The area under the ROC curve(AUC) was from 0.8854 to 0.9062 for the GFPLA-specific metabolite biomarker panel (**Figure 6f**).

### Correlation analysis of clinical laboratory indicators, differential metabolites and relative abundance of *Klebsiella* genus

To further explore the relationship among clinical manifestations, metabolic conditions within the abscess cavity and pathogenic bacteria, we conducted a correlation analysis of clinical laboratory indicators, the top 5 differential metabolites, and the relative abundance of *Klebsiella* genus(the most prevalent pathogen in the abscess cavity), and the results were shown in **Supplementary Figure S3**. Correlation analysis suggested that all the differential metabolites were positively correlated with blood glucose and CRP levels with all P-values < 0.01. And there was no correlation between the differential metabolites and the relative abundance of *Klebsiella*. The blood glucose level was positively correlated to the relative abundance of *Klebsiella* and the CRP level(P both < 0.05). The relative abundance of *Klebsiella* was also positively correlated to the CRP level(P < 0.05).

## Discussion

The results demonstrated that GFPLA patients had worse liver function, more severe inflammatory responses, and a higher in-hospital mortality rate than non-GFPLA patients, in line with previous studies indicating that GFPLA has more severe clinical characteristics (Thng et al., 2018; Zhang et al., 2021; Alzibdeh et al., 2023; Chen et al., 2023; Cui et al., 2023). Investigations of risk factors associated with GFPLA and microorganisms and metabolic characteristics within the abscess cavity can help clarify the underlying pathological mechanisms of the greater disease severity in patients with GFPLA and explore better treatment strategies. However, there are few reports on this aspect.

It has been reported about the close relationship between GFPLA and DM (Lee et al., 2010; Thng et al., 2018; Alzibdeh et al., 2023; Chen et al., 2023). Lee et al. hypothesized that gas formation in PLA is linked to increased gas production, impaired gas transportation, and an impaired equilibrium between gas in local tissues and that in abscesses (Lee et al., 2004), and all of these conditions can be effectively created by DM. The immune system of patients with diabetes might be less robust as that of healthy individuals, making them more susceptible to bacterial infections and reducing their ability to eliminate bacteria, which leads to abscess formation in the liver (Lin et al., 2004; Hoshi et al., 2023; Wang et al., 2023; Joshi et al., 2024). The high glucose levels in the tissues of patients with DM provide a favorable microenvironment for vigorous microbe metabolism and growth (Rayfield et al., 1982; Huang et al., 1991), which may lead to more gas production. Furthermore, patients with DM often have microvascular complications (Zhang et al., 2023; Meir et al., 2024), leading to insufficient blood supply to tissues and impaired clearance of metabolic waste, resulting in the decreased transport and accumulation of gas produced within the abscess cavity. The progression of PLA considered to be linked to gas formation in PLA, but the study results did not identify the time from symptom onset to CT as an independent risk factor for GFPLA.

In the conventional bacterial culture results, positivity for *K. pneumoniae* and *E. coli* was not identified as an independent risk factor for GFPLA, which contrasts with conventional views. Compared to conventional bacterial culture, 16S rDNA sequencing offers higher accuracy and a more comprehensive detection of pathogenic bacteria. Therefore, it was performed to further investigate the correlation between the bacterial community within the abscess cavity and GFPLA. The results indicated that alpha diversity, bacterial composition and microbiome phenotypes were similar between the two groups. Several rare GFPLA cases caused by gas-forming bacteria such as *C. perfringens* and *S. enteritidis* have been reported (Tee Yu et al., 2017; Hoshi et al., 2023; Tohmatsu et al., 2023). However, these bacteria were not detected in any of the 55 pus samples. The above results did not reveal any correlation between gas formation and the pathogenic bacteria composition in the pus cavity, and it might be partly because the main pathogen, *Klebsiella* inhibits the influence of other genera of bacteria. Previous studies have reported that *Klebsiella* may inhibit the growth of other microorganisms or even kill them through mechanisms such as competing for nutrients and increasing virulence (Kienesberger et al., 2022; Osbelt et al., 2024; Tan et al., 2024).

Untargeted metabolomics indicated that there were significant metabolic differences between the two groups. Among them, possible lac−phe, glu gln, 6−(gamma,gamma−dimethylallylamino)purine, N−lactoylleucine, and tyrosylglycine are five of the metabolites with the most significant differences, which are all positively correlated with blood glucose levels and CRP levels, but have no correlation with the relative abundance of the *Klebsiella*. Therefore, we speculate that high blood glucose levels may promote the formation of GFPLA by regulating the metabolism in the liver microenvironment rather than the relative abundance of pathogenic bacteria, thus leading to a more severe level of inflammation. Biochemically, hyperglycemia disturbs amino acid metabolism, causing abnormal changes in amino - acid - related metabolites like possible lac−phe, N−lactoylleucine, Glu gln, and tyrosylglycine; these abnormal metabolite levels create a more favorable growth environment for bacteria, providing abundant carbon and nitrogen sources (Heianza et al., 2019; Iatcu et al., 2021; Li et al., 2024). 6−(gamma, gamma−dimethylallyl amino)purine, a purine - type metabolite, may play a role in bacterial energy metabolism and nucleic acid synthesis (Wu et al., 2020; Kasahara et al., 2023). In addition, these differential metabolites exhibit good predictive performance for GFPLA, which offer new perspectives for the treatment of GFPLA: we can inhibit the formation of GFPLA by regulating specific metabolic pathways or targeting specific metabolites, thereby alleviating the clinical symptoms of patients and improving their prognosis.

This study had some limitations. First, this was a single-center study, and multicenter studies are needed to increase the sample size in the future. Second, we didn’t quantitatively detect and qualitatively analyze the gases in the abscess cavities. Finally, we only conducted a correlation analysis among the differential metabolites within the abscess cavity, the relative abundance of pathogenic bacteria, and clinical indicators, and did not perform experiments to verify the relevant speculations and further investigate the underlying mechanism of the formation and pathogenic mechanism of GFPLA.

## Conclusion

GFPLA have more serious clinical manifestations than non-GFPLA. DM is an independent risk factor for GFPLA. Gas formation could be related to high blood glucose levels and the metabolism in the liver microenvironment, but no correlation was found between the gas-formation and pathogen composition in the pus cavity in the current study.

## Data Availability

The datasets presented in this study can be found in online repositories. The names of the repository/repositories and accession number(s) can be found below: PRJNA917077 (SRA) and MTBLS7128 (Metabolights).
