# Supplementary material for: Clinical, microbial and metabolic characteristics of gas-forming pyogenic liver abscess and its potential formation mechanism
Source: Front Cell Infect Microbiol. 2026 Feb 11;16:1649624. doi: 10.3389/fcimb.2026.1649624 (PMC12932461; doi:10.3389/fcimb.2026.1649624)
Supplement: Supplementary file 1 [file Table1.docx]

**Material and method description of 16SrDNA sequencing**

**DNA extractions**

DNA from pus samples was extracted using the E.Z.N.A. ®Stool DNA Kit(D4015, Omega, Inc., USA) according to manufacturer ’s instructions. The reagent which was designed to uncover DNA from trace amounts of sample has been shown to be effective for the preparation of DNA of most bacteria. Nuclear-free water was used for blank. The total DNA was eluted in 50 μL of Elution buffer and stored at -80 °C until measurement in the PCR.

**PCR amplification and 16S rDNA sequencing**

| Region | Primers |
| --- | --- |
| V3-V4^1^ | 341F (5'-CCTACGGGNGGCWGCAG-3')  805R (5'-GACTACHVGGGTATCTAATCC-3') |
| V4^2^ | 515F(5'-GTGYCAGCMGCCGCGGTAA-3')  806R (5'- GGACTACHVGGGTWTCTAAT-3') |
| V4-V5 | F(5’-GTGCCAGCMGCCGCGG-3’)  R(5’-CCGTCAATTCMTTTRAGTTT-3’) |
| Archae^3^ | F(5’-GYGCASCAGKCGMGAAW-3’)  R(5’-GGACTACHVGGGTWTCTAAT-3’) |

The 5' ends of the primers were tagged with specific barcods per sample and sequencing universal primers. PCR amplification was performed in a total volume of 25 μL reaction mixture containing 25 ng of template DNA, 12.5 μL PCR Premix, 2.5 μL of each primer, and PCR-grade water to adjust the volume. The PCR conditions to amplify the prokaryotic 16S fragments consisted of an initial denaturation at 98 ℃ for 30 seconds; 32cycles of denaturation at 98 ℃ for 10 seconds, annealing at 54℃ for 30 seconds, and extension at 72 ℃ for 45 seconds; and then final extension at 72 ℃ for 10 minutes. The PCR products were confirmed with 2% agarose gel electrophoresis. Throughout the DNA extraction process, ultrapure water, instead of a sample solution, was used to exclude the possibility of false-positive PCR results as a negative control. The PCR products were purified by AMPure XT beads (Beckman Coulter Genomics, Danvers, MA, USA) and quantified by Qubit( Invitrogen, USA). The amplicon pools were prepared for sequencing and the size and quantity of the amplicon library were assessed on Agilent 2100 Bioanalyzer (Agilent, USA) and with the Library Quantification Kit for Illumina (Kapa Biosciences, Woburn, MA, USA), respectively. The libraries were sequenced on NovaSeq PE250 platform.

# Data analysis

Samples were sequenced on an Illumina NovaSeq platform according to the manufacturer's recommendations, provided by LC-Bio. Paired-end reads was assigned to samples based on their unique barcode and truncated by cutting off the barcode and primer sequence. Paired-end reads were merged using FLASH. Quality filtering on the raw reads were performed under specific filtering conditions to obtain the high-quality clean tags according to the fqtrim(v0.94). Chimeric sequences were filtered using Vsearch software(v2.3.4). After dereplication using DADA2,we obtained feature table and feature sequence. Alpha diversity and beta diversity were calculated by normalized to the same sequences randomly. Then according to SILVA(release 132) classifier, feature abundance was normalized using relative abundance of each sample . Alpha diversity is applied in analyzing complexity of species diversity for a sample through 5 indices, including Chao1, Observed species, Goods coverage, Shannon, Simpson, and all this indices in our samples were calculated with QIIME2. Beta diversity were calculated by QIIME2,the graphs were drew by R package. Blast was used for sequence alignment, and the feature sequences were annotated with SILVA database for each representative sequence. Other diagrams were implemented using the R package(v3.5.2).

**Material and method description of untargeted metabolomics analysis**

**Metabolite extraction recipes**

a) The collected samples were thawed on ice;

b) Add 500 μL precooled 80% methanol to 1.5mL EP tube;

c) 50 mg sample was added to the tube and homogenized;

d) The extraction mixture was then stored at -20°C for 2 h;

e) After centrifugation at 20,000 *g* for 10 min, the supernatants were transferred into new 1.5mL EP tube and dried;

f) The dried extract was stored at -80°C;

g) Reconstitute the dried extract in 100 μL precooled 80% methanol, take an equal part of each sample as polled QC sample.

After extracting metabolite, first group samples to ensure that different groups are cross-sorted on the machine，all samples were acquired by the LC-MS system followed machine orders. All chromatographic separations were performed using a Thermo Scientific UltiMate 3000 HPLC. An ACQUITY UPLC T3 column (100 mm*2.1 mm, 1.8 µm, Waters, UK) was used for reversed-phase separation. The column oven was maintained at 50°C. The flow rate is 0.3 mL/min, and the mobile phase consists of phase A (water, 0.1% formic acid) and phase B (acetonitrile, 0.1% formic acid). Gradient elution conditions were set as follows: 0～0.8 min, 2% B; 0.8～2.8 min, 2% to 70% B; 2.8~5.6 min, 70% to 90% B; 5.6~8 min, 90% to 100% B;8～8.1 min, 100% to 2% B; 8.1～10 min, 2% B. The injection volume for each sample was 4 µl. First scan one or two WASH samples, followed by 3-4 QCs, then insert a QC for every 10 samples scanned, and finally insert 2 QCs.

Then a high-resolution tandem mass spectrometer Q-Exactive (Thermo Scientific) was used to collect first and secondary order spectrum data of the metabolites eluted form the column. The Q-Exactive was operated in both positive and negative ion modes. Precursor spectra (70–1050 m/z) were collected at 70,000 resolution to hit an AGC target of 3e6. The maximum inject time was set to 100 ms. A top 3 configuration to acquire data was set in DDA mode. Fragment spectra were collected at 17,500 resolution to hit an AGC target of 1e5 with a maximum inject time of 50 ms. Fragmentation energy (stepped nce) is set as: 20, 40, 60 eV. Ion source (ESI) parameter settings: spray voltage (|KV|)) was 4000 (positive ion mode) and 4000 (negative ion mode), Sheath gas flow rate was 35, Aux gas flow rate was 10, Capillary temperature was 320℃.

Then import the acquired mass spectrum raw data (.raw files) into Compound Discoverer 3.1.0 (Thermo Fisher Scientific, USA) for data pretreatments, including: peak extraction, retention time correction within and between groups, adduct ion merging , fill gaps, background peak labeling and metabolite identification. Each ion was identified by combining retention time (RT) and m/z data. Intensities of each peaks were recorded and finally export information such as feature molecular weight, retention time, peak area and identification results.The online KEGG, HMDB database was used to annotate the metabolites by matching the exact molecular mass data 、name and formula of samples with those from database. If a mass difference between observed and the database value was less than 10 ppm, the metabolite would be annotated.

The intensity of peak data was further preprocessed by metaX. Those features that were detected in less than 50% of QC samples or 80% of biological samples were removed, the remaining peaks with missing values were imputed with the k-nearest neighbor algorithm to further improve the data quality. PCA was performed for outlier detection and batch effects evaluation using the pre-processed dataset. Probabilistic Quotient Normalization (PQN) was used to normalize the data to obtain the normalized ion intensity data of each sample。Quality control-based robust LOESS signal correction was fitted to the QC data with respect to the order of injection to minimize signal intensity drift over time. In addition, the coefficient of Variation（CV）of the metabolic features were calculated across all QC samples, and those > 30% were then removed.

Student t-tests were conducted to detect differences in metabolite concentrations between 2 phenotype. The P value was adjusted for multiple tests using an FDR (Benjamini-Hochberg). Supervised PLS-DA was conducted through metaX to discriminate the different variables between groups and VIP value was calculated. A VIP cut-off value of 1.0 was used to select important features. KEGG enrichment analysis was performed on significantly different metabolites (satisfying ratio >= 2 or ratio <= 1/2 & p value <= 0.05 & VIP>1).

Supplementary Figure 1: Correlation analysis among pathogenic bacterial at the genus level.
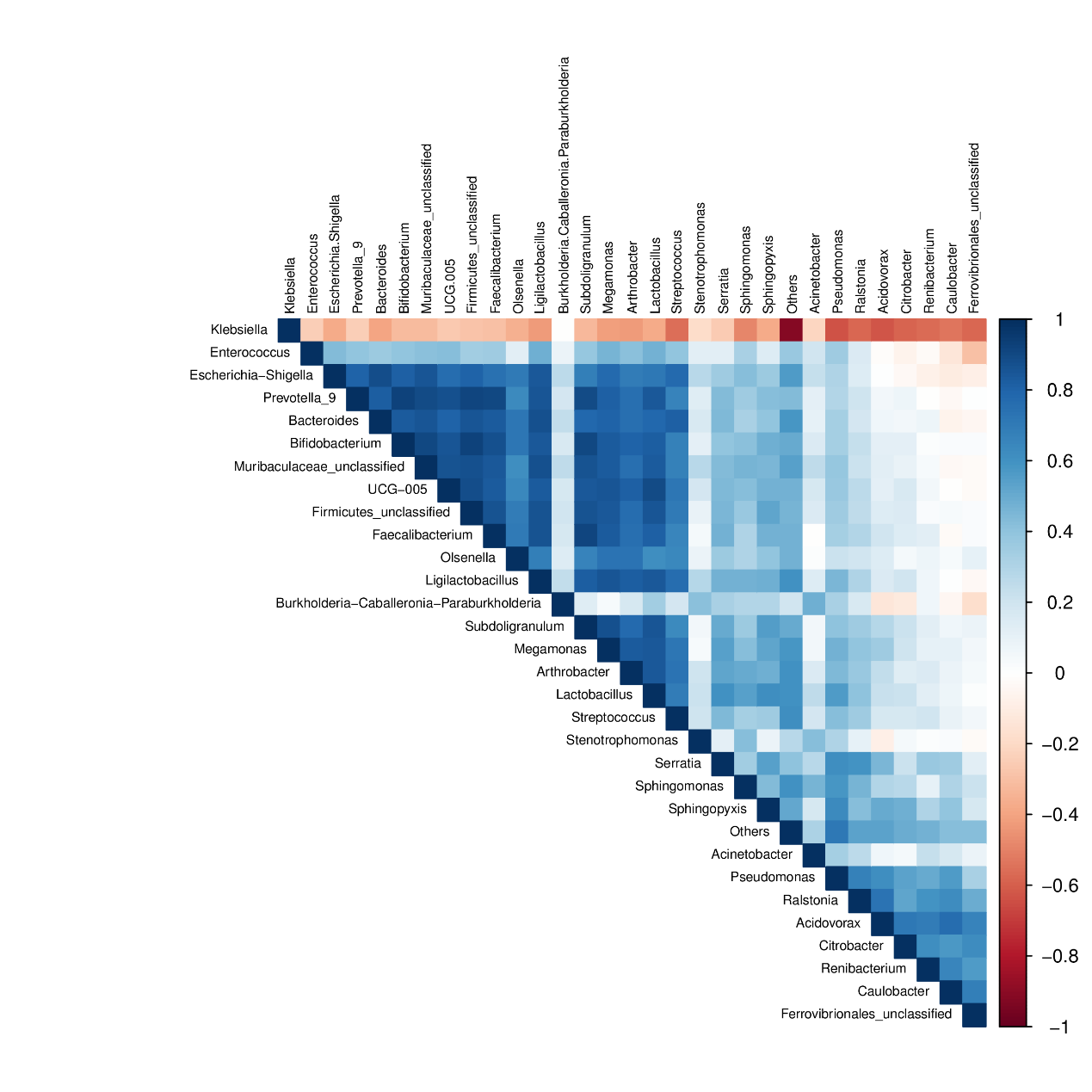


Supplementary Figure 2: Several metabolic pathways and functional enrichment analysis using PICRUSt program.


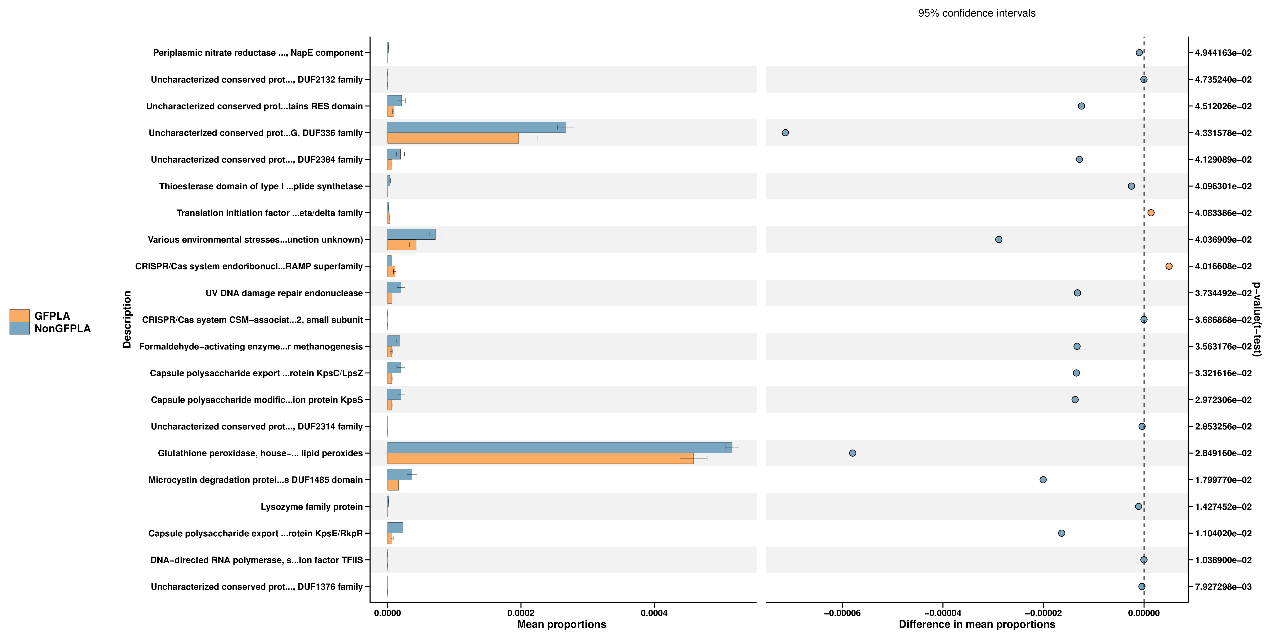


Supplementary Figure 3: Correlation analysis among clinical laboratory indicators, differential metabolites and relative abundance of Klebsiella genus.


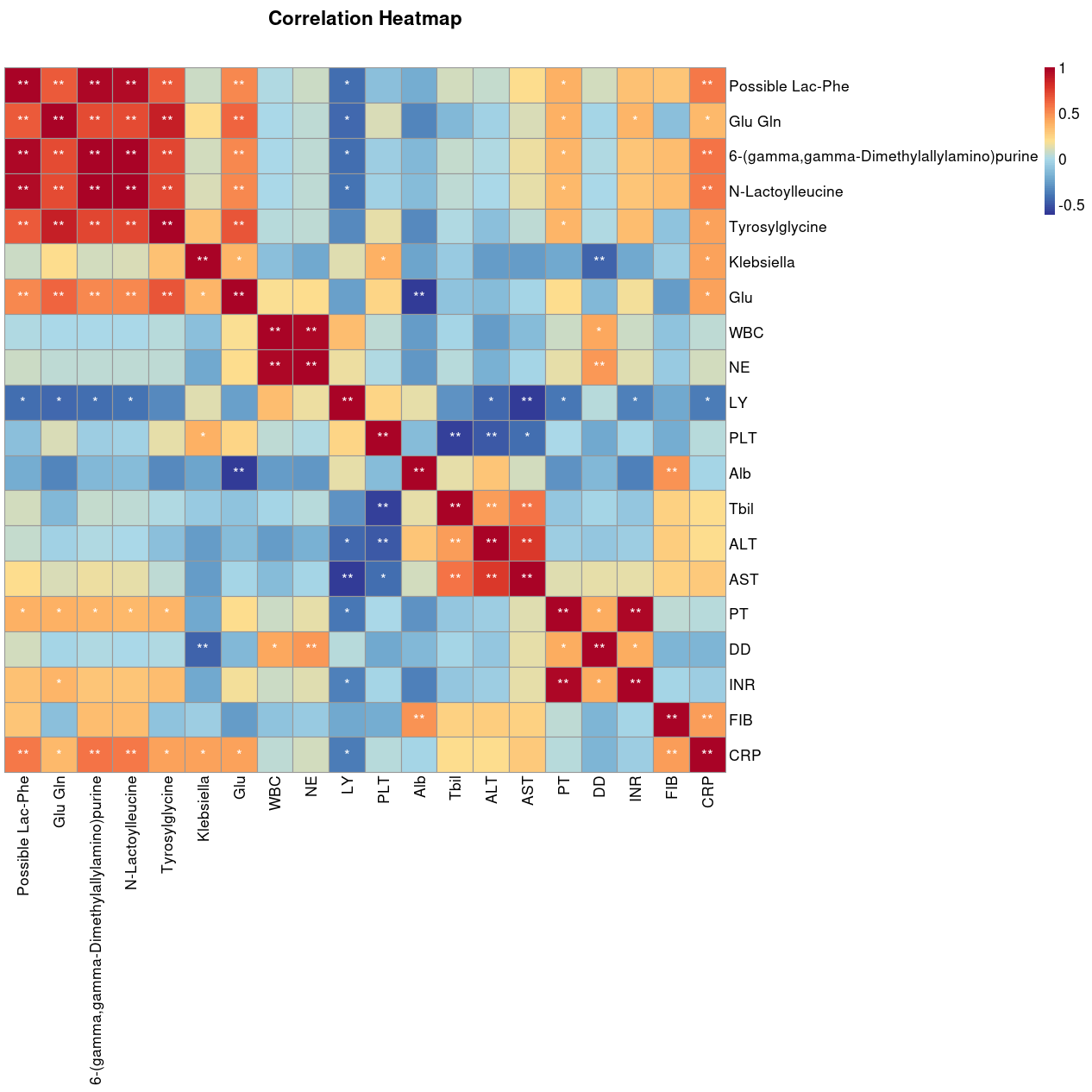


**Reference**：

1. Logue JB, Stedmon CA, Kellerman AM, Nielsen NJ, Andersson AF, Laudon H, et al. Experimental insights into the importance of aquatic bacterial community composition to the degradation of dissolved organic matter. *Isme j.* 2016;10(3):533-545.

2. Walters W, Hyde ER, Berg-Lyons D, Ackermann G, Humphrey G, Parada A, et al. Improved Bacterial 16S rRNA Gene (V4 and V4-5) and Fungal Internal Transcribed Spacer Marker Gene Primers for Microbial Community Surveys. *mSystems.* 2016;1(1).

3. Takai K, Horikoshi K. Rapid detection and quantification of members of the archaeal community by quantitative PCR using fluorogenic probes. *Appl Environ Microbiol.* 2000;66(11):5066-5072.
